# Color-coded summation images in the evaluation of renal artery stenosis before and after percutaneous transluminal angioplasty

**DOI:** 10.1186/s12880-020-00540-w

**Published:** 2021-02-10

**Authors:** Anne Marie Augustin, Stefan Welsch, Thorsten Alexander Bley, Kai Lopau, Ralph Kickuth

**Affiliations:** 1 Department of Diagnostic and Interventional Radiology, University Hospital Würzburg, Würzburg, Germany; 2 Department of Internal Medicine, Division of Nephrology, University Hospital Würzburg, Würzburg, Germany

**Keywords:** Digital subtraction angiography, Color-coded, Endovascular, Renal artery, PTA

## Abstract

**Background:**

Endovascular therapy is the gold standard in patients with hemodynamic relevant renal artery stenosis (RAS) resistant to medical therapy. The severity grading of the stenosis as well as the result assessment after endovascular approach is predominantly based on visible estimations of the anatomic appearance. We aim to investigate the application of color-coded DSA parameters to gain hemodynamic information during endovascular renal artery interventions and for the assessment of the procedures´ technical success.

**Methods:**

We retrospectively evaluated 32 patients who underwent endovascular renal artery revascularization and applied color-coded summation imaging on selected monochromatic DSA images. The differences in time to peak (dTTP) of contrast enhancement in predefined anatomical measuring points were analyzed. Furthermore, differences in systolic blood pressure values (SBP) and serum creatinine were obtained. The value of underlying diabetes mellitus as a predictor for clinical outcome was assessed. Correlation analysis between the patients´ gender as well as the presence of diabetes mellitus and dTTP was performed.

**Results:**

Endovascular revascularization resulted in statistically significant improvement in 4/7 regions of interest. Highly significant improvement of perfusion in terms of shortened TTP values could be found at the segmental artery level and in the intrastenotical segment (p < 0.001), significant improvement prestenotical and in the apical renal parenchyma (p < 0.05). In the other anatomic regions, differences revealed not to be significant. Differences between SBP and serum creatinine levels before and after the procedure were significant (p = 0.004 and 0.0004). Patients´ gender as well as the presence of diabetes mellitus did not reveal to be predictors for the clinical success of the procedure. Furthermore, diabetes and gender did not show relevant correlation with dTTP in the parenchymal measuring points.

**Conclusions:**

The supplementary use of color-coding DSA and the data gained from parametric images may provide helpful information in the evaluation of the procedures´ technical success. The segmental artery might be a particularly suitable vascular territory for analyzing differences in blood flow characteristics. Further studies with larger cohorts are needed to further confirm the diagnostic value of this technique.

## Introduction

Renal artery stenosis (RAS) describes the pathologic narrowing of the renal artery by at least 50%, leading to a compromised blood supply to the kidney. RAS most commonly is a result of atherosclerotic vessel alterations originating from the aorta. As a consequence, the proximal segment of the renal artery including the ostium is predominantly affected [1]. Less frequent underlying conditions include fibromuscular dysplasia, congenital disorders and posttherapeutic alterations like previous surgery or radiation therapy. The long-term results of RAS may be resistant arterial hypertension, ischemic nephropathy with renal dysfunction and cardiac insufficiency [2].

Beside clinical and laboratory examinations, different diagnostic procedures are used for the work-up of RAS. In the diagnostic work-up, renal duplex ultrasound (US) is a well-established and rapidly performable non-invasive imaging method for both screening as well as follow-up examinations. Nevertheless, duplex US is highly dependent on the examiner skills and can be impeded by patients´ body habitus. Alternatively, cross-sectional imaging like computed tomography angiography (CTA) or magnetic resonance angiography (MRA) revealed high sensitivity and specificity rates in the assessment of RAS [3–5]. Performance of non-invasive diagnostic assessment prior to invasive management might be useful in providing information regarding anatomical variants and characteristics of the stenosis. However, since many patients are restricted by renal function, application of iodinated or gadolinium-based contrast media might be unfavourable due to the risk of contrast-induced nephropathy or nephrogenic systemic fibrosis (NSF), respectively, even if current studies mitigated the risk of NSF following standard doses of gadolinium-based contrast [6]. Minimally-invasive renal digital subtraction arteriography (DSA) is usually performed to confirm the diagnosis of RAS, simultaneously followed by endovascular therapy. The evaluation of monochromatic DSA series is predominantly based on the visual assessment of vessel morphology and contrast flow, and thus, subject of relevant interobserver variability.

On the contrary, color-coded DSA enables visualization of hemodynamic flow parameters within a single image. Concerning this post-processing approach, data has only been reported in the setting of neurovascular interventions and peripheral artery disease so far [7–9]. At the same time, information concerning the utilization of this tool in the endovascular treatment of RAS is lacking. Therefore, the aim of this study was to evaluate the benefits of color-coded parametric DSA images prior to and after endovascular RAS treatment with special regard to the mean time to peak (TTP) as a reliable parameter of technical success.

## Materials and methods

### Study cohort

A retrospective review of the archives of our interventional radiology division yielded the cases of 59 patients who consecutively underwent endovascular treatment of RAS between February 2009 and April 2019. Due to a lack of available DSA serial imaging and exclusive storage of single elected DSA images, 27 patients were excluded from the study, resulting in a final cohort of 32 patients (16 women, 16 men; median age 71.0 years; range 23–83 years).

All patients were examined and treated as part of routine care. Patients´ characteristics, clinical symptoms, comorbidities as well as diagnostic and therapeutic management strategies were extracted from the medical records. The local institutional review board approved this retrospective study and waived the need for written informed consent (Waiver No. 20200904 01). Written informed consent for the intervention was obtained from all patients prior to the procedure.

Underlying condition for RAS was atherosclerosis in the majority of cases (29 cases, 90.6%). Fibromuscular dysplasia was the underlying disease in two cases and stenosis of anastomosis of renal transplant graft in one further patient. In the last case, time between the renal transplantation and the endovascular procedure was 198 months. Clinical characteristics of the study cohort are summarized in Table 1.

**Table 1:** Clinical characteristics

	n	%
Median age (years)	71 (23—81)	
Male:female ratio	16:16	
*Risk factors*		
Diabetes mellitus	15	46.9
History of smoking	6	18.8
Dyslipidaemia	20	62.5
Obesity	8	25.0
COPD	3	9.4
Atherosclerosis	24	75.0
Coronary heart disease	8	25.0
Cardial insufficiency	11	34.4
*Non-invasive investigation*		
MRA	23	71.9
CTA	2	6.3
Duplex US	4	12.5
*Underlying condition*		
Atherosclerosis	29	90.6
FMD	2	6.3
Anastomoses stenosis	1	3.1
*Treatment side*		
Right	10	31.3
Left	12	37.5
Both	9	28.1
Renal transplant	1	3.1

*COPD* chronic obstructive pulmonary disease, *MRA* magnetic resonance angiography, *CTA* computed tomography angiography, *FMD* fibromuscular dysplasia

### Radiologic investigation

Aside from clinical and laboratory assessment, diagnosis of RAS based on non-invasive radiologic investigation including MRA in 24, CTA in two and duplex US in four cases. In two cases documented imaging was not available prior to the intervention, and the indication for the procedure was mainly based on clinical and laboratory findings.

### Procedure and image acquisition

All procedures were performed by the same operator in our local angiography suite (Axiom Artis Zee, Siemens AG, Healthcare Sector, Forchheim, Germany). Patients were advised not to discontinue antihypertensive medication. With all patients under local anaesthesia, endovascular procedures were carried out via a right retrograde femoral artery approach with a short unbraided or long braided 6-F to 7-F introducer sheath (Radifocus or Destination, Terumo, Japan), respectively. Due to the patients’ limited renal function, non-selective abdominal aortographies were not conducted. Instead, selective angiographies of the renal artery stenosis were acquired with manual injection of iodinated contrast (Imeron 300; Bracco Imaging, Konstanz, Germany) using selective catheters (Cobra- or VS-shaped, Cook Medical, Bjaeverskov, Denmark or Boston Scientific, Galway, Ireland).

All renal artery stenoses were passed utilizing a 0.014- or 0.018-inch guidewire (Cruiser 14, Biotronik, Bülach, Switzerland; Pointer, Argon Medical Devices, Frisco, TX). In two patients with FMD, balloon angioplasty was exclusively performed. In all cases of atherosclerotic RAS, pre-dilatation was conducted using a low-profile PTA-balloon (3–5 mm in diameter; Pacific, Medtronic, Parkway, MN or Armada 14, Abbott Vascular Redwood City, CA) with subsequent implantation of a balloon-expandable cobalt-chrome or bare metal stent (5–8 mm in diameter; 12–18 mm in length; Tsunami, Terumo, Tokyo; Herculink-Elite, Abbott Vascular, Redwood City, CA; Visipro, Medtronic, Parkway, MN; Formula; Cook Medical, Bjaeverskov, Denmark; Palmaz Blue, Cordis, Fremont, CA). Percutaneous transluminal angioplasty with stent implantation ended with angiographic documentation of an improvement of lumen gain and antegrade flow into the renal artery documented by manual injection of contrast media.

### Generation of color-coded images and data evaluation

After the procedure, acquired DSA series were sent to the local picture archiving and communication system (PACS) (Syngo Plaza®, Siemens Healthcare GmbH, Erlangen, Germany and Merlin, Phoenix PACS®, Freiburg, Germany).

Post-processing of the monochromatic to polychromatic images was performed utilizing the commercially available software Syngo iFlow® (Siemens Healthcare GmbH, Erlangen, Germany), running at a dedicated system software WinNT 5.2, SP 2 at a specialized working station (Syngo XWP, Siemens Healthcare GmbH, Erlangen, Germany).

This tool enables the visualization of blood flow information extracted from a monochromatic DSA series within one single image using color-coding [10]. In this context, the algorithm calculates the time between contrast injection and reaching the maximum of opacification for each image pixel. Blood flow is than expressed by flow curves using different colors starting with dark blue, indicating delayed blood flow up to red representing high flow velocities. Furthermore, quantitative blood flow parameters can be generated for any region of interest, such as an area under the curve and a mean time to peak.

Measurement of the DSA series were performed in a standardized manner. In this context, DSA series before and after endovascular treatment of RAS were selected, in which the selective catheter or long introducer sheath was positioned within or near the ostium of the renal artery. Thus, the renal artery was visible in its full length including the renal parenchyma. Only cases with available DSA images fulfilling the criteria described above were included.

**Fig. 1:**
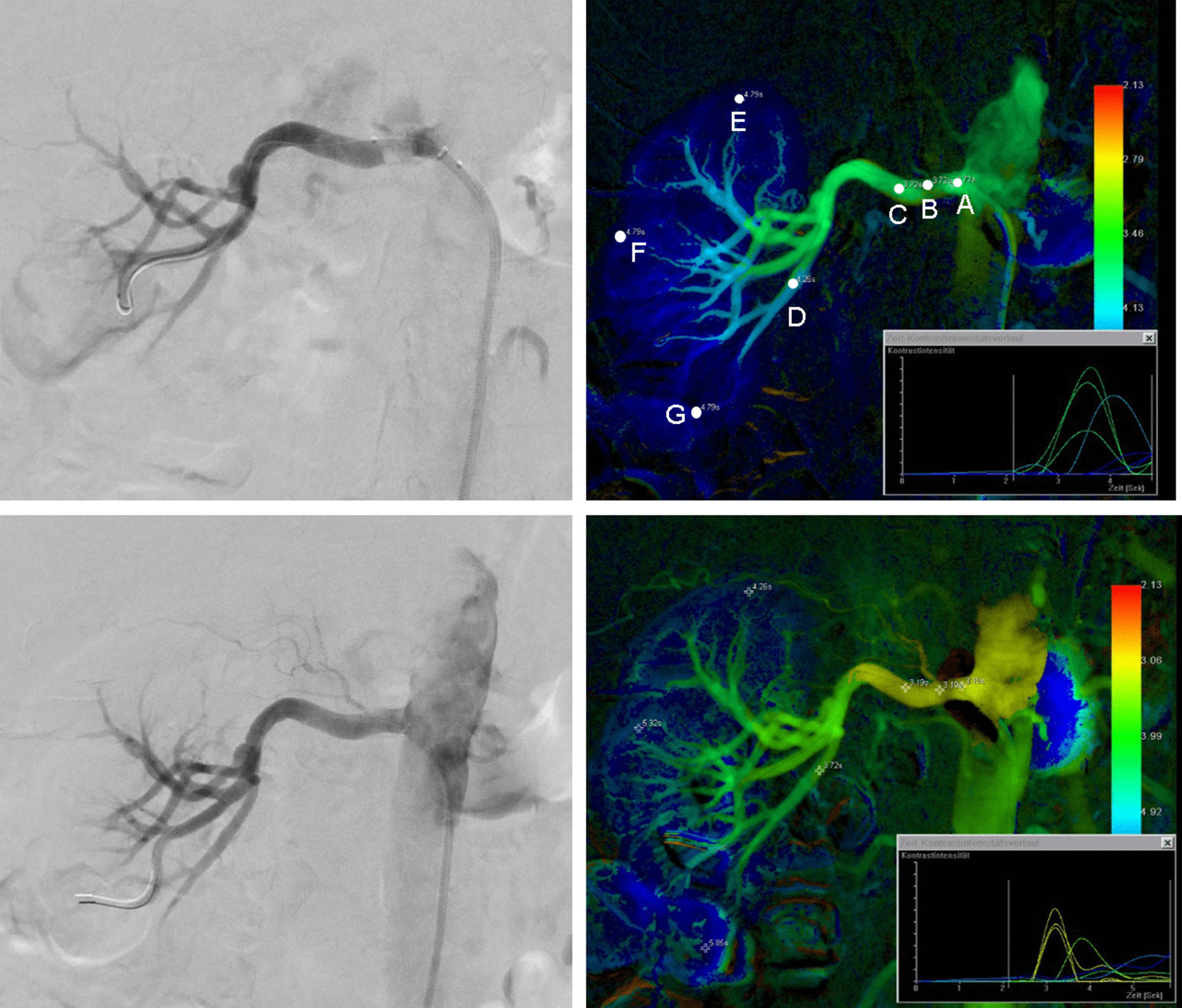
Monochromatic and corresponding color-coded images before and after renal artery revascularization. A Selective angiography of the right renal artery demonstrates significant proximal atherosclerotic stenosis. B Corresponding parametric image with TTP values in the predefined regions of interest (ROIs; A = prestenotic, B = intralesional, C = poststenotic, D = segmental artery of first order at the caudal pole, E = apical renal parenchyma, F = renal parenchyma at the level of hilus, G = renal parenchyma at the caudal pole. C After PTA and stent implantation monochromatic DSA does not reveal residual stenosis. D Color-coded summation image with the same ROIs demonstrates accelerated blood flow within the renal artery close to the former stenosis and within the segmental artery of first order at the caudal pole. Warmer color gradient also indicates better blood supply to the kidney

**Fig. 2:**
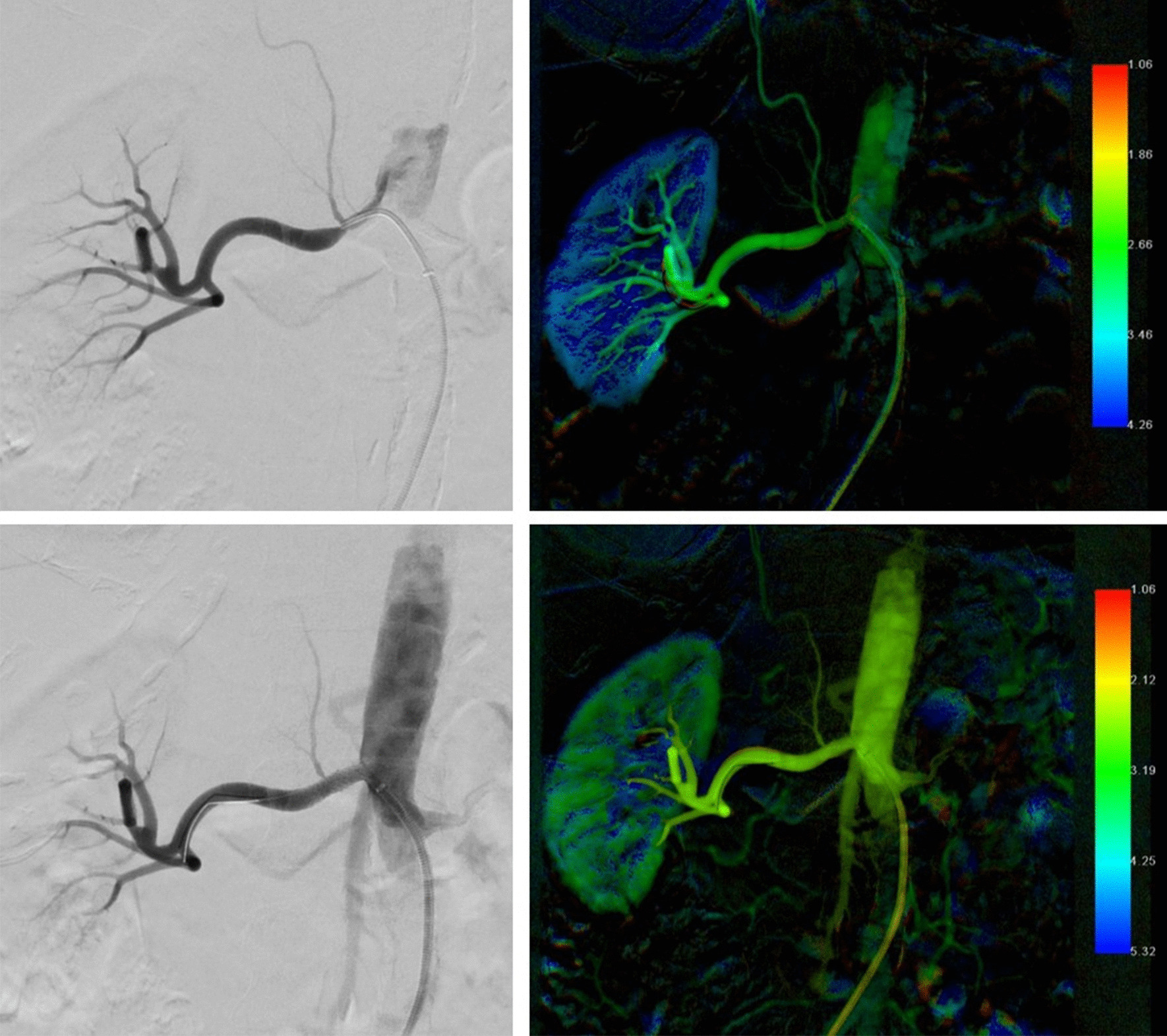
Monochromatic and color-coded DSA images before and after PTA with stent implantation. **a** Selective angiography of the right renal artery shows ostial stenosis. **b** Corresponding color-coded image reveals a slight color-inhomogeneity within the proximal renal artery, indicating relevant flow-limitation. **c** After PTA and stent implantation monochromatic DSA does not reveal any residual stenosis. **d** Corresponding parametric DSA shows homogenous color-coding within the renal artery after stenting and a warmer color-gradient, indicating improved blood flow

After application of the color-coded algorithm, seven standardized measurement points were manually determined as a region of interest (ROI) for each parametric single image (Figs. 1, 2). Those measuring points included:

- renal artery proximal to the stenosis or abdominal aorta at the level of the origin of the renal artery (prestenotic),
- intralesional,
- renal artery distal to the stenosis (poststenotic),
- segmental artery of first order at the caudal pole,
- apical renal parenchyma,
- renal parenchyma at the level of hilus,
- renal parenchyma at the caudal pole.

The TTP (in seconds) was measured in all the mentioned ROIs in the DSA series before and after angioplasty of the renal artery. The differences of the TTP (dTTP) in the single ROIs pre- and postinterventionally were assessed in order to gain information concerning changes in blood flow characteristics and thus serving as a parameter of technical success. Blood pressure and serum creatinine values were analyzed before and after the intervention. Median follow-up was 24 days and ranged between 1 and 848 days. Blood pressure measurement was performed as non-invasive assessment using an arm-cuff. Correlation between the dTTP and blood pressure as well as creatinine parameters were analyzed in order to estimate the predictive value of color-coded parameters for the clinical outcome of the patients. Being a known risk factor for atherosclerosis and development of RAS, correlation between the presence of diabetes mellitus and the differences in TTP at the parenchymal level was also evaluated. Gained data was collected with Excel (Microsoft Office 365 ProPlus, Version 1803).

### Statistical analysis

Statistical analysis was performed using a dedicated software (R®, Version 4.0.2, The R Foundation for Statistical Computing, Wien, Austria). Descriptive data were presented as means ± standard deviation (SD) for normally distributed variables or medians with ranges for non-normally distributed variables, if appropriate; categorical data were expressed as counts and percentages with n (%). With regard to assessment of normality, the Shapiro test was used rejecting the hypothesis of normality when the p-value was less or equal to 0.05.

TTP values, blood pressure and creatinine before and after the procedure were compared using Wilcoxon´s signed rank test in cases of non-normally distributed variables and Student´s T test in normally distribution of variables. Differences in TTP values (on the level of segmental artery) were compared with differences of creatinine levels utilizing the Spearman-test. The Mann–Whitney-U-test or T-test was used to analyze whether presence of diabetes mellitus and patients´ gender might have influenced the procedures’ outcome. In this context, possible predictors for the procedures´ outcome were aimed to be found.

## Results

In total, 32 RAS were treated by endovascular revascularization and assessed using color-coding algorithm prior to and after the procedure. All cases demonstrated noticeable improvement of antegrade flow following PTA with or without stent implantation, resulting in a technical success rate of 100%.

**Fig. 3:**
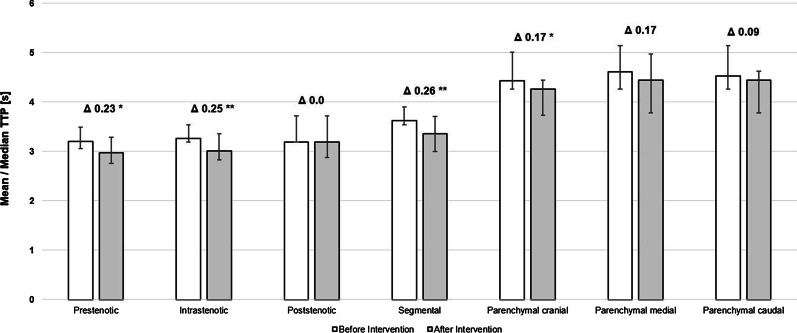
Mean and median TTP before and after endovascular revascularization including the 25/75% quantile, dTTP and its statistical significance (p < 0.05*; p < 0.01**; p < 0.001***)

**Fig. 4:**
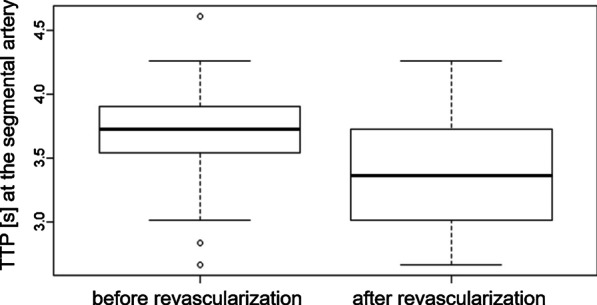
PTT in seconds before and after revascularization of the renal artery at the level of the segmental artery

Mean and median TTP evaluation before and after renal artery revascularization in the seven predetermined measurement points revealed significant reduction of TTP and thus improvement of blood flow in 4/7 assessed regions (Fig. 3). Highly significant differences in the TTP could be found within the treated stenosis and within the first segmental artery at the caudal pole (p < 0.001) (Fig. 4). Statistically significant improvement of the TTP were also documented within the renal artery distal to the stenosis and within the apical renal parenchyma (p < 0.05). Within the other measuring regions, differences were not statistically significant (p > 0.05). Detailed results are shown in Table 2.

**Table 2:** Results of systolic blood pressure, serum creatinine values, as well as TTP values from color-coded DSA images in the predefined ROIs before and after the procedure

	Mean/median		Delta	p value
	Prior to intervention	After intervention		
Systolic blood pressure [mmHg]	169	140	29	0.0004***
Serum creatinine [ml/dl]	2.473	1.400	1.073	0.004**
*TTP [s]*				
Prestenotic	3.200	2.974	0.226	0.018*
Intrastenotic	3.261	3.007	0.254	0.008**
Poststenotic	3.190	3.190	0.000	0.064
Segmental	3.625	3.361	0.264	0.009**
Parenchymal cranial	4.430	4.260	0.170	0.037*
Parenchymal medial	4.610	4.440	0.170	0.073
Parenchymal caudal	4.525	4.440	0.085	0.420

**Fig. 5:**
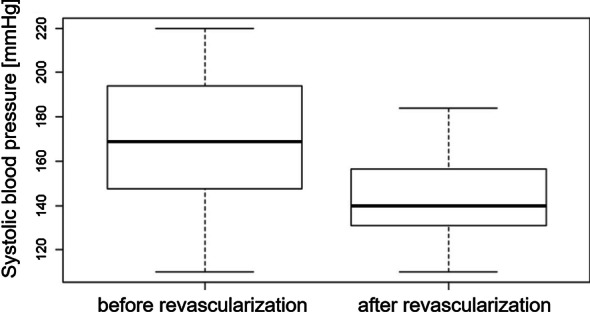
Systolic blood pressure values (mmHg) before and after endovascular treatment of the renal artery stenosis

Evaluation of creatinine levels of the treated patients revealed highly significant reductions after endovascular renal artery treatment (p = 0.004). Blood pressure measurements resulted in highly significant reductions after endovascular revascularization (p = 0.0004; Fig. 5).

Since dTTP values revealed to be highest in the segmental artery level, a correlation analysis was performed regarding this measurement point as representative for all values. In this context, a statistically significant correlation between the dTTP and the difference of creatinine levels was not found (p = 0.472). Moreover, correlation between dTTP and improvement of blood pressure values were not statistically significant (p = 0.292).

A significant influence of the patients´ gender on the differences in blood pressure (p = 0.836) or on changes of creatinine values (p = 0.316) owing to endovascular procedure was not found. Thus, gender did not reveal to be a predictor for the clinical outcome. Furthermore, existing diabetes mellitus was not a predictor for improvement of blood pressure (p = 1.0) and creatinine values (p = 0.486).

The correlation analysis of prevalence of diabetes and patients´ gender, respectively, and the changes of TTP (dTTP) in the measuring points within the renal parenchyma did not show a significant correlation (p-values ranging between 0.299 and 0.750).

## Discussion

Due to long-term effects and comorbidities, especially with severe renal impairment, RAS is associated with increased mortality rates [11]. The main treatment goals of patients with RAS include blood pressure adjustment, improvement of renal function and control of fluid shifts. First-line therapy remains pharmaceutical, with optimal medical therapy involving blood pressure control, lipid lowering and antiplatelet therapy. In cases of hemodynamically significant RAS with resistant hypertension after aggressive pharmaceutical treatment, invasive therapy might be indicated [12]. In this context, surgical revascularization techniques like endarterectomy or bypass surgery have increasingly been replaced by endovascular approaches [13].

Uncertainty about the right therapy regime was exacerbated by large clinical randomized trials like ASTRAL and CORAL, stating no superiority of renal artery revascularization compared with medical therapy with respect to the prevention of clinical adverse events [14, 15]. Nevertheless, both trials are subject of debate and controversy due to the relevant selection bias and shortcomings in study design [16]. In contrast, the benefit of renal artery revascularization in terms of a decrease in blood pressure and creatinine levels in patients with critical renal function has been shown in different observational studies [17, 18]. In this context, renal artery stenting might be able to delay worsening of renal function [19]. Moreover, while being characterized by high technical success rates, overall complication rates are low [20].

In the assessment of RAS, non-invasive MRI-based methods to evaluate the hemodynamic significance of RAS in a porcine model revealed a good correlation between the non-invasive transstenotic pressure gradient derived from unenhanced MRI-imaging and endovascular pressure gradient obtained from DSA [21, 22]. However, study results showed a tendency for overestimation of the stenosis in MRI. In addition, the utilization of this technique in a larger patient population with exclusive RAS has not been investigated yet. As another MRI-based approach, functional contrast-enhanced MR perfusion measurement revealed to be feasible in RAS grading and the gained parameters correlated well with serum creatinine levels [23]. On the other hand, phase-contrast MRI (PC-MRI) is a non-invasive imaging method visualizing blood flow velocity with no need for contrast agent application. It´s high reproducibility and correlation with other hemodynamic measurement techniques has recently been confirmed by a meta-analysis [24]. The avoidance of iodine-containing contrast agent and radiation exposure must be mentioned as an advantage of all MRI-based imaging methods. Nevertheless, those imaging studies are more beneficial in the context of pre-treatment evaluation and to assess the mid- and long-term outcome after revascularization but can not be applied intraprocedurally.

In general, the availability of cross-sectional imaging like MRI and CT studies before the intervention is desirable, since it might provide beneficial information, for example regarding the vascular access and the presence of hard- and softplaques in the vascular segments to be treated, and thus may have a positive impact on the development of the endovascular strategy. In the presented study cohort, most patients received CT or MRI examinations prior to the procedure. Nevertheless, in cases of a very high suspicion for RAS based on clinical signs and duplex sonographic findings, further imaging studies had been bypassed and the indication for the procedure without further screening was at the discretion of the referring nephrologist and performing interventional radiologist, as suggested by current guidelines [25].

In contrast, the periprocedural assessment of the interventions´ technical success is mainly based on the visual evaluation of the monochromatic DSA series to date. However, visual assessment of a stenosis severity on the basis of angiography remains subjective and has been shown to correlate poorly with objective measurable parameters like translesional arterial pressure gradients with calculation of renal fractional flow reserve (FFR) [26]. Those functional parameters might thus be a helpful tool for further characterization of a lesions´ hemodynamic relevance. Nevertheless, so far, a consensus does not exist, which pressure gradient parameters and thresholds should be used in evaluating a lesion´s significance. Furthermore, results of pressure measurements might be influenced by the catheter placement with a relevant risk of distortion [27]. Consequently, it seems worthwhile to create and implement further diagnostic tools that might possibly enable an objective monitoring of the procedures´ technical outcome. In this study, we report our experiences with the utilization of parametric DSA images in the assessment of the technical success after endovascular revascularization in RAS and identified the mean TTP as a solid parameter. Color-coded angiography provides quantitative information by an easy and quick-available post-processing mode of conventional DSA images. As a consequence, further administration of contrast agent or radiation exposure is not necessary. Being a novel technique, data regarding the diagnostic benefit of parametric DSA is limited.

In a most recently published study, the direct comparison between color-coded single images and conventional monochromatic DSA in the evaluation of superficial femoral artery lesions after percutaneous angioplasty did not reveal any superiority of the parametric imaging mode in an ROC analysis [28]. Nevertheless, color-coded images might be particularly beneficial as an adjunctive diagnostic tool that, together with the conventional monochromatic assessment, may improve diagnostic quality. The application of this post-processing technique in the assessment of renal artery stenosis, including the attempt of a post-procedural success monitoring of the performed treatment, has not been reported elsewhere so far.

Since the final monochromatic angiography showed improvement of the anatomical vessel diameter by visual assessment in our study and the results also revealed a significant improvement of mean and median TTP in four out of seven predefined measurement points, an interrelation between those two effects might be worth considering. Accordingly, the study of Tan et al. showed a statistically significant correlation between the changes of anatomic degree of stenosis and the dTTP of inflow stenosis in haemodialysis accesses, while a correlation analysis with respect to the outflow lesions did not revealed a significant correlation [29]. The authors hypothesized that those differences might be caused by variations of outflow components in the assessed haemodialysis accesses.

While our results demonstrated improved mean and median TTP values in most of the measured anatomic locations as a result of the intervention, the highest effect was documented at the level of the segmental artery. In contrast, TTP parameters at the level of renal parenchyma revealed little influence caused by the treatment. One possible explanation for this observation might be chronic vascular alterations resulting from long-term ischemia in patients with RAS. Those modifications are likely to be permanent and the possible influence by revascularization techniques might be restricted or at least only measurable on a long-term basis. Furthermore, correct assessment of blood flow values at subsegmental artery levels might be limited due to vessel size, also representing a technical obstacle. Even if the intrastenotic and poststenotic dTTP appeared to be significant, these measuring points should be put into question, since the proper measurement in this region might be technically difficult to implement. Major reasons might be a complex stenosis morphology and the small residual vessel lumen, possibly resulting in inaccurate results. Additionally, given by the definition of the TTP, the overall hemodynamic effect of a stenotic lesion might only be shown adequately distal to the stenosis.

Based on our results, we hypothesize that the level of the renal segmental artery is the most suitable anatomical vessel region for assessing hemodynamic changes after revascularization. As a possible compatible hemodynamic correlate, the so called “tardus-parvus-pattern” is a known phenomenon in the doppler sonographic assessment of RAS that can be observed in the vessel region distal to the stenosis. This pattern at the downstream circulation of a stenotic lesion is caused by a decreased magnitude of blood flow within the ventricular systole due to the vessel narrowing [30].

Being a potential indicator for the response of therapy, blood pressure measurements and serum creatinine were evaluated before and after the procedure and revealed significant improvements. At the same time, a correlation between patients´ gender and the extent of the effect of clinical therapy was not found. Furthermore, the presence of diabetes mellitus did not correlate with an improvement of the systolic blood pressure or creatinine level. Moreover, a significant influence of the presence of diabetes mellitus and dTTP in the peripheral renal segments could not be found. The hypothesis behind this analysis was the presumption that renal microangiopathy, which is a known long-term sequela in patients with diabetes mellitus, might impact the TTP values by the procedure [31]. Thus, based on our results, the analyzed patients´ specifics did not prove to be suitable predictors for the procedures´ clinical success.

There are several limitations of the presented study. First, the heterogeneity of the included patients results in differences in the patients´ risk profile as well as the chosen therapeutic management. Thus, patients with underlying fibromuscular dysplasia were treated with solely balloon PTA of the affected renal artery, while most of the patients with atherosclerotic RAS were treated by a combination of balloon PTA and stent implantation. Furthermore, patients with atherosclerotic alterations leading to RAS are generally characterized by older age and a higher number of comorbidities. Second, follow-up of the patients was not standardized, consecutively the time between the underlying procedure and the assessment of post-interventional parameters varied significantly. Since the impact of RAS on the blood pressure and the renal function is complex and effects of long-term existing RAS might not be completely reversible or at least require a certain amount of time, a uniform and sufficiently long follow-up period might have led to different and more robust results. This might also explain the missing correlation between the clinical therapy responses in blood pressure and serum creatinine with the technical success indicated by TTP. Third, the study design is retrospective, and the number of included patients is limited. Thus, further investigations with a larger patient cohort and a prospective design should be aimed for in order to generate more robust and transferable data. Fourth, parameters of manual contrast agent application before and after angioplasty were not standardized which theoretically may result in deviations that influence the color-coded images. Finally, due to the absence of patients with different procedural outcome in terms of technical failure, the assessment of color-coding DSA usefulness remains limited. As there is no comparison of TTP in patients who did and did not undergo the procedure, including final outcome or prognosis, therefore the added value of this parameter as a diagnostic tool can not be proven by the available data. Therefore, a study design taking those prerequisites into account would be desirable to adequately address the topic of clinical relevance.

## Conclusions

In conclusion, data and color maps obtained from parametric DSA algorithm provide immediate hemodynamic functional information during endovascular renal artery revascularization and may help to evaluate the procedures’ technical success. The level of the segmental artery seems to be the most suitable anatomic region regarding assessment of blood flow characteristics.

## Data Availability

The date used and analyzed during the current study are available on reasonable request.

## Abbreviations

AUC: Area under the curve
COPD: Chronic obstructive pulmonary disease
CTA: Computed tomography angiography
DSA: Digital subtraction angiography
FMD: Fibromuscular dysplasia
PTA: Percutanous transluminal angioplasty
MRA: Magnetic resonance angiography
RAS: Renal artery stenosis
PACS: Picture archiving and communication system
POBA: Plain old balloon angioplasty
PSVR: Peak systolic velocity ratio
ROC: Receiver operating characteristics
TTP: Time to peak

## References

[1] Kaatee R, Beek FJ, Verschuyl EJ, vd Ven PJ, Beutler JJ, van Schaik JP (1996). Atherosclerotic renal artery stenosis: ostial or truncal?. Radiology.

[2] Tafur JD, White CJ (2017). Renal artery stenosis: when to revascularize in 2017. Curr Probl Cardiol.

[3] Turgutalp K, Kiykim A, Ozhan O, Helvaci I, Ozcan T, Yildiz A (2013). Comparison of diagnostic accuracy of Doppler USG and contrast-enhanced magnetic resonance angiography and selective renal arteriography in patients with atherosclerotic renal artery stenosis. Med Sci Monit.

[4] Tan KT, van Beek EJ, Brown PW, van Delden OM, Tijssen J, Ramsay LE (2002). Magnetic resonance angiography for the diagnosis of renal artery stenosis: a meta-analysis. Clin Radiol.

[5] Kim TS, Chung JW, Park JH, Kim SH, Yeon KM, Han MC (1998). Renal artery evaluation: comparison of spiral CT angiography to intra-arterial DSA. J Vasc Interv Radiol.

[6] Weinreb JC, Rodby RA, Yee J, Wang CL, Fine D, McDonald RJ, et al. Use of intravenous gadolinium-based contrast media in patients with kidney disease: consensus statements from the American College of Radiology and the National Kidney Foundation. Radiology. 2020:202903. 10.1148/radiol.2020202903 33170103

[7] Kostrzewa M, Kara K, Pilz L, Mueller-Muertz H, Rathmann N, Schoenberg SO (2017). Treatment evaluation of flow-limiting stenoses of the superficial femoral and popliteal artery by parametric color-coding analysis of digital subtraction angiography series. Cardiovasc Intervent Radiol.

[8] Lin CJ, Luo CB, Hung SC, Guo WY, Chang FC, Beilner J (2013). Application of color-coded digital subtraction angiography in treatment of indirect carotid-cavernous fistulas: initial experience. J Chin Med Assoc.

[9] Golitz P, Struffert T, Lucking H, Rosch J, Knossalla F, Ganslandt O (2013). Parametric color coding of digital subtraction angiography in the evaluation of carotid cavernous fistulas. Clin Neuroradiol.

[10] Strother CM, Bender F, Deuerling-Zheng Y, Royalty K, Pulfer KA, Baumgart J (2010). Parametric color coding of digital subtraction angiography. AJNR Am J Neuroradiol.

[11] Mui KW, Zeebregts CJ, van den Hout H, van Baal JG, Navis G, Jan-Woittiez A (2011). Impact of incidental renal artery stenosis on long-term mortality in patients with peripheral arterial disease undergoing vascular procedure. J Vasc Surg.

[12] Mousa AY, Bates MC, Broce M, Bozzay J, Morcos R, AbuRahma AF (2017). Issues related to renal artery angioplasty and stenting. Vascular.

[13] Goldfarb DA (2005). Increase in utilization of percutaneous renal artery interventions by medicare beneficiaries, 1996–2000. J Urol.

[14] Cooper CJ, Murphy TP, Cutlip DE, Jamerson K, Henrich W, Reid DM (2014). Stenting and medical therapy for atherosclerotic renal-artery stenosis. N Engl J Med.

[15] Investigators A, Wheatley K, Ives N, Gray R, Kalra PA, Moss JG (2009). Revascularization versus medical therapy for renal-artery stenosis. N Engl J Med.

[16] Mohan IV, Bourke V (2015). The management of renal artery stenosis: an alternative interpretation of ASTRAL and CORAL. Eur J Vasc Endovasc Surg.

[17] Raman G, Adam GP, Halladay CW, Langberg VN, Azodo IA, Balk EM (2016). Comparative effectiveness of management strategies for renal artery stenosis: an updated systematic review. Ann Intern Med.

[18] Iannone LA, Underwood PL, Nath A, Tannenbaum MA, Ghali MG, Clevenger LD (1996). Effect of primary balloon expandable renal artery stents on long-term patency, renal function, and blood pressure in hypertensive and renal insufficient patients with renal artery stenosis. Cathet Cardiovasc Diagn.

[19] Prince M, Tafur JD, White CJ (2019). When and how should we revascularize patients with atherosclerotic renal artery stenosis?. JACC Cardiovasc Interv.

[20] White CJ (2007). Catheter-based therapy for atherosclerotic renal artery stenosis. Prog Cardiovasc Dis.

[21] Bley TA, Francois CJ, Schiebler ML, Wieben O, Takei N, Brittain JH (2016). Non-contrast-enhanced MRA of renal artery stenosis: validation against DSA in a porcine model. Eur Radiol.

[22] Bley TA, Johnson KM, Francois CJ, Reeder SB, Schiebler ML (2011). Noninvasive assessment of transstenotic pressure gradients in porcine renal artery stenoses by using vastly undersampled phase-contrast MR angiography. Radiology.

[23] Michaely HJ, Schoenberg SO, Oesingmann N, Ittrich C, Buhlig C, Friedrich D (2006). Renal artery stenosis: functional assessment with dynamic MR perfusion measurements–feasibility study. Radiology.

[24] Villa G, Ringgaard S, Hermann I, Noble R, Brambilla P, Khatir DS (2020). Phase-contrast magnetic resonance imaging to assess renal perfusion: a systematic review and statement paper. MAGMA.

[25] Martin LG, Rundback JH, Wallace MJ, Cardella JF, Angle JF, Kundu S (2010). Quality improvement guidelines for angiography, angioplasty, and stent placement for the diagnosis and treatment of renal artery stenosis in adults. J Vasc Interv Radiol..

[26] Subramanian R, White CJ, Rosenfield K, Bashir R, Almagor Y, Meerkin D (2005). Renal fractional flow reserve: a hemodynamic evaluation of moderate renal artery stenoses. Catheter Cardiovasc Interv.

[27] Nahman NS, Maniam P, Hernandez RA, Falkenhain M, Hebert LA, Kantor BS (1994). Renal artery pressure gradients in patients with angiographic evidence of atherosclerotic renal artery stenosis. Am J Kidney Dis.

[28] Augustin AM, Thein I, Rickert N, Klink T, Bley TA, Kickuth R (2020). Evaluation of superficial femoral artery-lesions after percutaneous transluminal angioplasty: color-coded summation images vs. monochromatic digital subtraction angiography. BMC Med Imaging..

[29] Tan RY, Chong TT, Tsai FC, Pang SC, Lee KG, Gogna A (2018). A pilot study on adjunctive use of parametric colour-coded digital subtraction angiography in endovascular interventions of haemodialysis access. BMC Med Imaging.

[30] Stavros AT, Parker SH, Yakes WF, Chantelois AE, Burke BJ, Meyers PR (1992). Segmental stenosis of the renal artery: pattern recognition of tardus and parvus abnormalities with duplex sonography. Radiology.

[31] Romero-Aroca P, Mendez-Marin I, Baget-Bernaldiz M, Fernendez-Ballart J, Santos-Blanco E (2010). Review of the relationship between renal and retinal microangiopathy in diabetes mellitus patients. Curr Diabetes Rev.

